# Conchological differentiation and genital anatomy of Nepalese Glessulinae (Gastropoda, Stylommatophora, Subulinidae), with descriptions of six new species

**DOI:** 10.3897/zookeys.675.13252

**Published:** 2017-05-23

**Authors:** Prem B. Budha, Fred Naggs, Thierry Backeljau

**Affiliations:** 1 University of Antwerp, Evolutionary Ecology Group, Universiteitsplein 1, B-2610, Antwerp, Belgium; 2 Natural History Museum, Cromwell Road, London, SW7 5BD, UK; 3 Central Department of Zoology, Tribhuvan University, Kirtipur, Kathmandu, Nepal; 4 Royal Belgian Institute of Natural Sciences, Vautierstraat 29, B-1000, Brussels, Belgium

**Keywords:** flagellum, genitalia, *Glessula*, identification key, Nepal, *Rishetia*, shell sculpture, spermatophore

## Abstract

Eleven species of Glessulinae belonging to the genera *Glessula* Martens, 1860 (three species) and *Rishetia* Godwin-Austen, 1920 (eight species) are reported from Nepal, six of which are new to science and are described here, viz., *G. tamakoshi* Budha & Backeljau, **sp. n.**, *R. kathmandica* Budha & Backeljau, **sp. n.**, *R. nagarjunensis* Budha & Naggs, **sp. n.**, *R. rishikeshi* Budha & Naggs, **sp. n.**, *R. subulata* Budha & Naggs and *R. tribhuvana* Budha, **sp. n.** and two are new records for Nepal viz. G. cf. hebetata and R. cf. mastersi. The relation between the shell height-width ratio and the structure of the proximal part of the male reproductive organs in Glessulinae is explored. Illustrations and a key for the identification of the Nepalese Glessulinae are provided, including the first record of a spermatophore in *Rishetia*.

## Introduction

The Glessulinae Godwin-Austen, 1920 are one of the most speciose achatinoid subfamilies (Gastropoda, Stylommatophora) with more than 160 nominal species. The majority of these are known from India and Sri Lanka (Nevill 1878, Beddome 1906, Pilsbry 1908-1909, Gude 1914, Godwin-Austen 1920). A few species occur in Myanmar and very few are recorded from Thailand, Vietnam, Borneo, Sumatra, and Java (Tenison-Woods 1888, Pilsbry 1908–1909, Panha 1995–1996, Schileyko 2011). Kashmir and Himachal Pradesh, India, form the western limit of glessulines in the Himalaya, where they are represented by only three species: *Glessula huegeli* (L. Pfeiffer, 1842), *G. paupercula* (Blanford, 1861) and *G. tornensis* Blanford, 1870 (Gude 1914, Surya Rao and Mitra 2005). In contrast, the eastern Himalaya, the NE Indian states of West Bengal, Sikkim, Assam, Arunachal Pradesh, Nagaland, Meghalaya, Mizoram and Manipur, including Nepal (east of Kaligandaki river) shows a much higher (n > 50) glessuline species diversity (Godwin-Austen 1920, Budha et al. 2015).

In what is widely consulted as the most recent review of gastropod classification, Bouchet and Rocroi (2005) accept the placement of *Rishetia* Godwin-Austen, 1920 as the type genus of the Rishetiinae Schileyko, 1999 within Subulinidae but do not follow Schileyko, 1999 in placing *Glessula* Martens, 1860 in a separate family, the Glessulidae Godwin-Austen, 1920. They place the Glessulinae within the Subulinidae. However, the generic status and relationship between *Glessula* and *Rishetia* remain unclear and *Rishetia* is often synonymised with *Glessula* (Zilch 1959, Mitra et al. 2005, Ramakrishna et al. 2010). Until this wide range of doubt is resolved, from synonymising *Rishetia* within *Glessula* to placing *Rishetia* in a distinct family, we provisionally follow Godwin-Austen (1920) in recognising *Rishetia* and *Glessula* as being closely related groups at generic level. This uncertainty can be largely attributed to the limited anatomical information on glessulids, the taxonomy of which is largely based on shells, and to the wide geographical range of species that have been included within *Glessula*. Outside of South Asia *Glessula* is known from shell characters only. Conversely, glessuline genital anatomy exhibits significant variation, but the genitalia of only a few species of this large group are currently documented (Semper 1877, Godwin-Austen 1918, 1920, Fernando 1950, Schileyko 1999). More generally, since Godwin-Austen’s (1920) work, only a few sporadic studies of glessulines have been published (Schileyko and Kuznetsov 1996, Schileyko 1999, Ramakrishna and Mitra 2002, Mitra et al. 2005, Surya Rao and Mitra 2005, Surya Rao et al. 2007, Raheem et al. 2008, 2014, Ramakrishna et al. 2010 and Budha et al. 2015).

To date, only four nominal glessuline species have been reported from Nepal, namely *Glessula subjerdoni* Beddome, 1906, *Glessula orobia* (Benson, 1860), *Rishetia tenuispira* (Benson, 1836) and *Rishetia hastula* (Benson, 1860) (Schileyko and Kuznetsov 1996, Schileyko 1999, Budha et al. 2015). In the present study we include data on the genital anatomy of 10 species, describe six new species and provide a dichotomous identification key to all currently known *Glessula* and *Rishetia* species in Nepal.

## Material and methods

**Figure 1.**
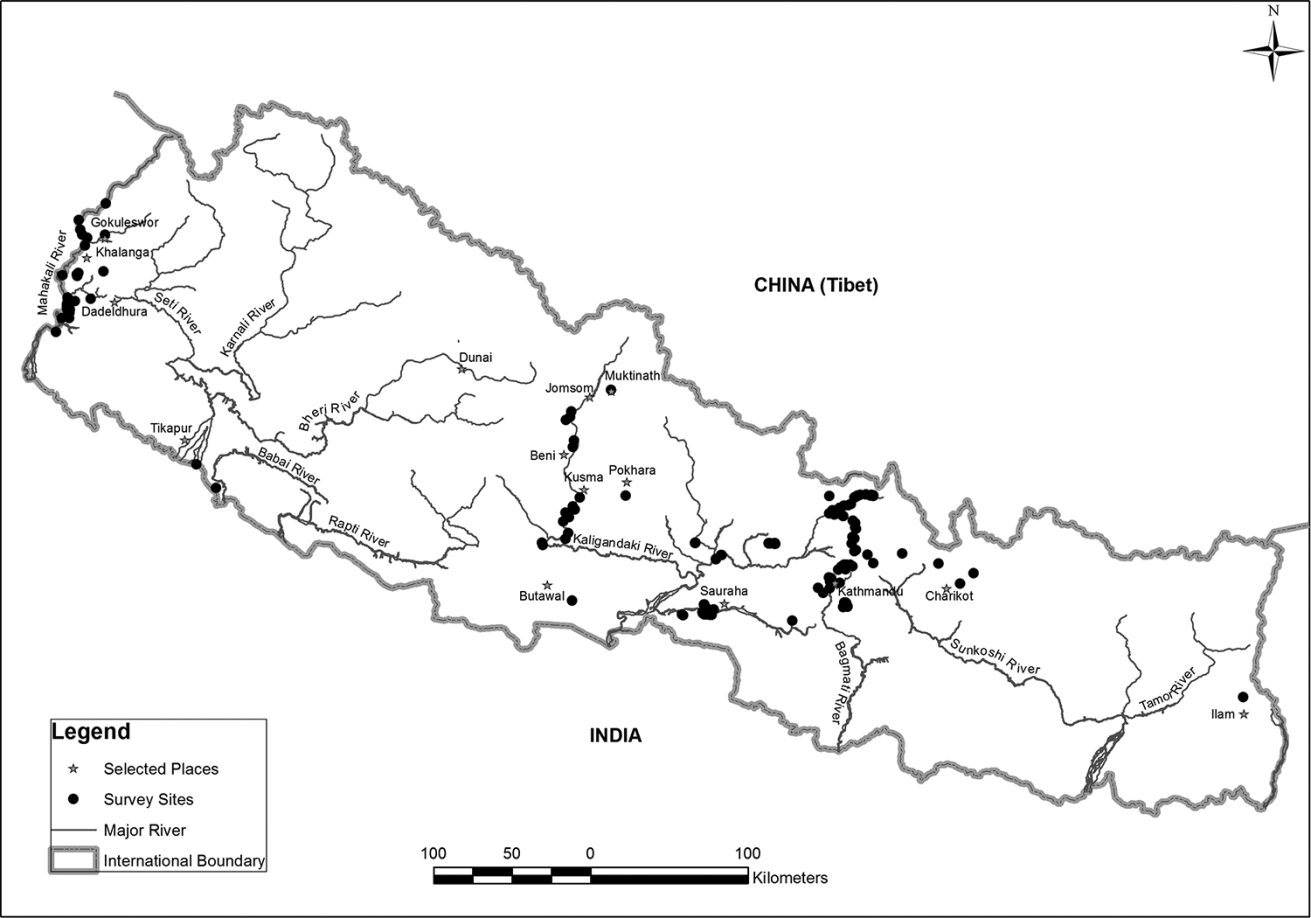
Collecting locations for glessulines in Nepal.

From 2006 to 2010 glessuline shells and specimens were hand-collected during surveys in the Baitadi, Bardiya, Dadeldhura, Darchula, Chitwan, Dolakha, Gulmi, Ilam, Kailali, Kathmandu, Lalitpur, Nawalparasi and Tanahun districts in Nepal, including three national parks: Shivapuri-Nagarjun National Park, Langtang National Park and Chitwan National Park. Collecting locations are shown in Figure 1.

Snails were drowned in water and preserved in 90% ethanol, which was changed at least once within a week after collection. All new material is deposited in the Central Department Zoology Museum of Tribhuvan University (CDZMTU), Nepal. The numbers of shells in the samples are indicated after the registration numbers. The letter ‘P’ indicates that the sample consists of live-collected specimens preserved in ethanol. Illustrations were made using a camera lucida mounted on a stereomicroscope. Features of the interior of the penis were drawn from photographs by Rajman Maharjan (Natural History Museum, Kathmandu). Whorls were counted and shells were measured (in mm) with electronic digital callipers as described by Kerney and Cameron (1979). All descriptions in this paper are based on material collected in Nepal.

We avoided the shell terms ‘protoconch’ and ‘teleoconch’ because the demarcation between these shell parts is unclear in glessulines. So we used the first whorl and second whorl for ‘protoconch’and the other whorls for ‘teleoconch’. The term ‘apical whorls’ is used for the first three or more whorls together. The terminology of the reproductive organs was modified from Tompa (1984). We arbitrarily differentiate the proximal parts of the male reproductive organs as ‘epiphallic caecum’, being the structure positioned close to where the vas deferens joins the epiphallus, and as ‘flagellum’, being the other sac-like structure connected to the epiphallus. The internal surface of the penis is referred to as smooth or folded to avoid ill-defined functional terms such as ‘stimulator’ and ‘pilaster’. In this paper we refer proximal part toward the free end of the male genitalia and the distal part closer toward genital orifice.

Type and other reference material was examined in the collections at the Natural History Museum, London (NHM), the University Museum of Zoology, Cambridge (UMZC), the Royal Belgian Institute of Natural Sciences, Brussels (RBINS) and the Zoological Museum of Moscow State University (ZMMU), Moscow, Russia.

### Abbreviations used

Genitalia: AG: Albumen gland. AT: Atrium. EC: Epiphallic caecum. EP: Epiphallus. F: Flagellum. GD: Gametolytic duct. GS: Gametolytic sac. HD: Hermaphrodite duct. IP: Interior of Penis. P: Penis. PR: Penial retractor muscle. SO: Spermoviduct. V: Vagina. VD: Vas deferens.

Shell measurements: HA: height of aperture. SH: shell height. SW: shell width. Wh: number of whorls. WA: width of aperture.

## Systematics

### Subulinidae P. Fischer & Crosse, 1877

#### Glessulinae Godwin-Austen, 1920

##### Glessula

Taxon classificationAnimaliaGastropodaSubulinidae

von Martens, 1860

###### Distribution.

India, Bangladesh, Sri Lanka, Nepal, China, Myanmar, Borneo, Sumatra, Java, Thailand and Vietnam (Gude 1914, Panha 1995-1996, Schileyko 2011).

###### Type species.

*Achatina ceylanica* L. Pfeiffer, 1845

###### Main characteristics.

Shell ovate-conic or turreted, glossy in general, with or without spiral lirae on the first 1-2 whorls, first whorl rounded, body whorl broad, columella truncated. Vagina shorter than penis. The proximal part of male reproductive organ varies with respect to the form of the flagellum, i.e. from a comb-like structure with many notches, to a hand-like structure with two or more finger-like processes (Figs 3–5).

##### Glessula orobia

Taxon classificationAnimaliaGastropodaSubulinidae

(Benson, 1860)

Figs 2A , 3

Achatina orobia B. : Benson 1860, p. 461.
Achatina orobia , Benson: Hanley and Theobald 1876, pl. 18, fig. 7.
Stenogyra (Glessula) orobia , Benson: Nevill 1878, p. 170.
Glessula orobia (Benson): Pilsbry 1909, p. 96.
Glessula orobia Benson: Gude 1914, p. 427.
Glessula orobia Bs.: Godwin-Austen 1920, p. 19.

###### Material examined.

CDZMTU055/10 shells and CDZMTU055P/2 specimens (dissected), Maipokhari, Ilam, *Cryptomeria* forest, 2100 m, 27.006944N, 87.93000E, 29.X.2010. leg. P.B. Budha. Glessula orobia var. major Godwin-Austen, 1920: Syntypes NHMUK, Reg. no. 1986020, 2 shells, Richila Peak, Sikkim. *G. orobia* (Benson, 1860): Syntypes NHMUK, Reg. no. 1946.10.16.82-83, 2 shells, Senchal, Darjeeling, India. *G. orobia* (Benson), RBINS (I.G. 10591), 2 shells, Darjeeling, India.

###### Type locality.

“Sinchul et Darjiling (alt. ped. 8500 et 7000, NE India)”.

###### Distribution.

Nepal and NE India (Godwin-Austen 1920, Kuznetsov and Schileyko 1997, Budha et al. 2015).

**Figure 2.**
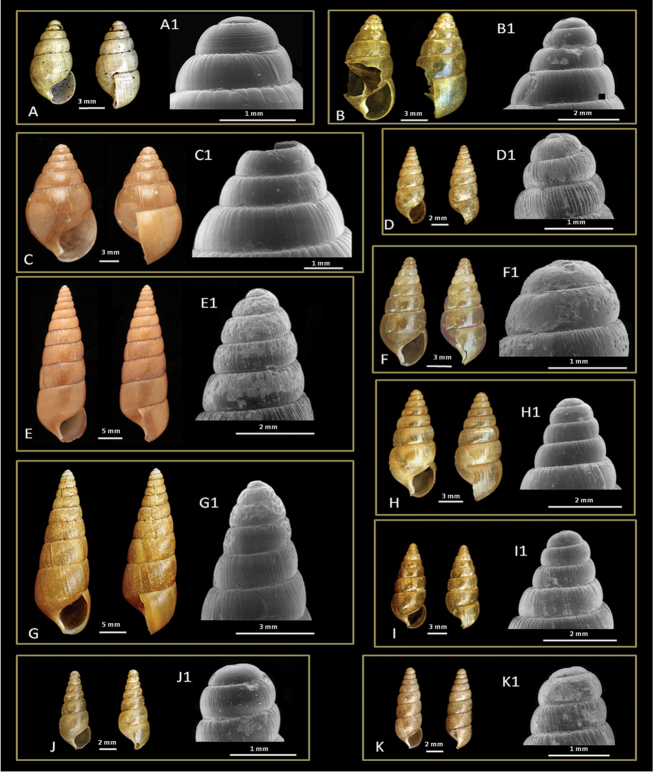
Shells (**A–K**) and SEM micrographs of apical whorls (**A1–K1**) of Nepalese glessulines. **A** *Glessula orobia* (Benson, 1860): CDZMTU055P, shell of dissected specimen, Maipokhari, Ilam, Eastern Nepal **B** Glessula cf. hebetata: CDZMTU056P, shell of dissected specimen, Godawari, Lalitpur, Central Nepal **C** *G. tamakoshi* sp. n., holotype: CDZMTU057P, shell of dissected specimen, Suridobhan, left bank of Tamakoshi River, Dolakha District, Central Nepal **D** *Rishetia hastula* (Benson, 1860): CDZMTU059P, shell of dissected specimen, Chitwan National Park, riverine forest opposite bank of Sauraha, Rapti River **E** *R. kathmandica* sp. n., holotype: CDZMTU062P, shell of dissected specimen, Godawari Botanical Garden, Lalitpur, Central Nepal **F** R. cf. mastersi: CDZMTU065P, shell of dissected specimen, Kurintar, Chitwan, degraded riverine bushes with big boulders, mixed *Shorea robusta* forest **G** *R. nagarjunensis* sp. n., holotype: CDZMTU067P, shell of dissected specimen, Nagarjun forest, Balaju-Jamacho trail Nagarjun-Shivapuri National Park, Kathmandu, Nepal **H** *R. rishikeshi* sp. n., holotype: CDZMTU0170P, shell of dissected specimen, Jhawalepakho Community Forest, Ridi, Gulmi District, montane hill *Shorea robusta* forest **I** *Rishetia* sp. CDZMTU078P, shell of dissected specimen, Boshikharka, Dhading, Central Nepal **J** *R. subulata* sp.n., holotype: CDZMTU072P, shell of dissected specimen, Godawari, along the Godawari-Phulchowki road approx. 200 m above the Naudhara Temple **K** *R. tribhuvana* sp. n., holotype: CDZMTU077, shell of dissected specimen, Tribhuvan University garden, Kirtipur, Kathmandu, Nepal.

**Shell**. Measurements (n = 6): SH 7.0–8.5 mm, SW 4.0–4.5 mm, HA 3.0–3.5, WA 2.0–2.5, Wh 6.0–7.0; approx. 1.8× higher than wide, thin, ovate-conic, fresh shells light yellowish, older shells straw coloured. Surface glossy, with widely spaced incised radial striations. The first whorl smooth, second whorl with 10–11distinct fine spiral lirae (Fig. 2A1), other whorls with widely spaced radial striations. Sides convex, suture impressed. Aperture nearly ovate, 1.7× higher than wide, margin simple and thick, columellar margin abruptly truncated, columella slightly curved.

###### Genitalia

**Figure 3.**
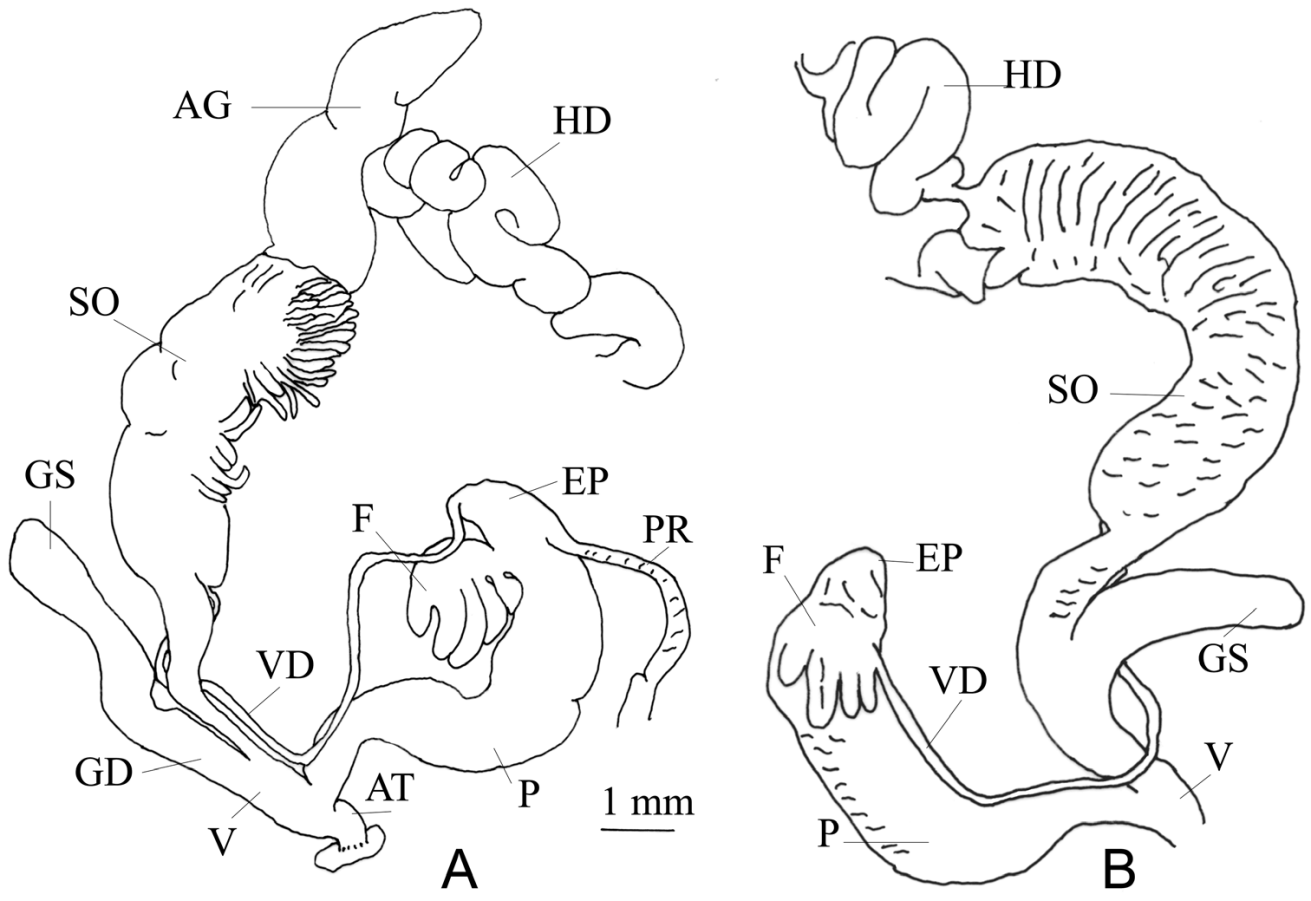
Genitalia of *G. orobia*: **A** General view, CDZMTU055P, Maipokhari, Ilam, Eastern Nepal **B** var. major reproduced from Godwin-Austen (1920), pl. CLXV, fig. 4.

(n = 2) (Fig. 3). Vas deferens with a constant diameter. The flagellum hand-shaped with five “fingers”. The first “finger” is small and pear-shaped, the fifth “finger” is comparatively short and positioned apart like a thumb (Fig. 3A). Penis cylindrical, basal portion narrower than the proximal portion. The diameters of the gametolytic sac and duct of the dissected specimens were not particularly different. The vagina short, nearly 1/4^th^ the length of the penis. The penial retractor muscle close to the flagellum. The albumen gland elongated, long, about half of the total length of the spermoviduct. The hermaphrodite duct is very thick.

###### Remarks.

Specimens were collected in eastern Nepal, at less than 30–40 km west of the type locality, Darjeeling, and at a similar altitude (7000 ft = 2100 m). Based on shell size Godwin-Austen (1920) distinguished var. major (SH 13.0, SW 5.2) and var. minor (SH 8.0–9.0, SW 3.75–4.0). He figured the genitalia of var. major from Damsang, Sikkim, with its hand-shaped flagellum (Fig. 3B) containing four finger-like processes, of which the first is short, while the second and the third are fused to a single finger. In contrast, the flagellum of Nepalese specimens has five distinct finger-like processes. Based on the shell size, Nepalese specimens belong to the var. minor. For the time being the taxonomic status of both varieties remains unclear.

##### Glessula cf. hebetata

Taxon classificationAnimaliaGastropodaSubulinidae

Godwin-Austen, 1920

Figs 2B , 4

Glessula hebetata : Godwin-Austen 1920, p. 49, pl. 162, fig. 26.

###### Material examined.

CDZMTU056/1 juvenile shell and CDZMTU056P/1 specimen (dissected), Godawari, approx. 150 m above the Godawari National Herbarium, Lalitpur, Central Nepal, 1636 m, 27.5965N, 85.3894E, 02.XII.2006, leg. P.B. Budha.

###### Type locality.

"Munipur" NE India.

###### Distribution.

Burrail range, Augaoluo Peak, Naga Hills, NE India; Nepal.

###### Shell.

Measurements (n = 1): SH 13.3 mm, SW 6.2 mm, HA 5.3 mm, WA 3.8 mm, Wh 7.0, approx. 2× higher than wide, solid, ovate-conic, yellowish. Surface, glossy, with widely spaced incised radial striations. The incised striations start from the first whorl, sides convex, suture shallow. Aperture semi-oval, 1.4× higher than wide, peristome simple and thick, columellar margin short and truncate.

###### Genitalia

**Figure 4.**
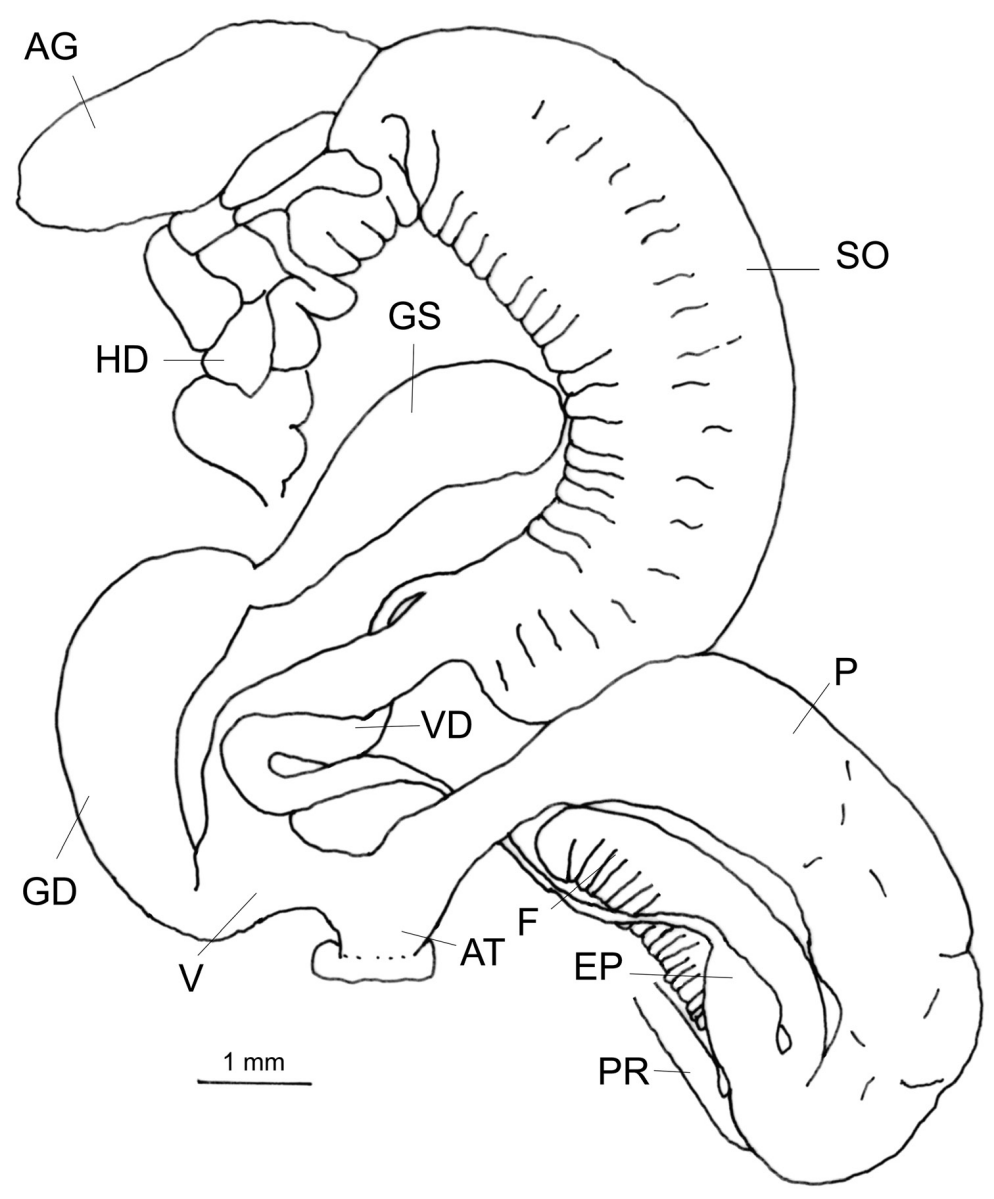
Genitalia of Glessula cf. hebetata: CDZMTU056P, Godawari, Lalitpur, Central Nepal.

(n = 1) (Fig. 4). Vas deferens widens towards the spermoviduct and opens into the terminal part of the small, pear shaped epiphallus. Flagellum comb-like with numerous notches in the comb, the terminal notch comparatively wider. The basal end of the penis cylindrical, widening from the middle to the proximal end (Fig. 4). Vagina very short, nearly 1/5^th^ of the length of the penis. The penial retractor muscle close to the flagellum. The gametolytic sac is elongated, connected to the gametolytic duct by a narrow neck. The convoluted mass of the hermaphroditic duct is thick and compact and the albumen gland in the dissected samples is short.

###### Remarks.

The sculpture, thick peristome and size of the Nepalese specimen match with the original description of *G. hebetata* Godwin-Austen, 1920 (p. 49, pl. 162, fig. 26). The genitalia suggest that G. cf. hebetata is closely related to *G. ochracea* Godwin-Austen, 1918, and *G. orophila* (Reeve, 1849), all sharing a similar comb-shaped flagellum. Yet, the shell of *G. ochracea* is larger (SH 21.25, SW 9.25) and shows a sharper striation (Godwin-Austen 1920). In contrast, with respect to *G. orophila*, Godwin-Austen (1920, p. 4) questioned the identity of the genitalia figured under this name by Semper (1873, pl. 12, fig.14–16). Hence, there is no other comparative data available with respect the genital anatomy of G. cf. hebetata, including of *G. hebetata* itself. However, the shell of the single specimen of putative *G. hebetata* from Nepal looks similar to the figure of Godwin-Austen (1920, p.49, pl. CLXII, fig. 26) which is why this specimen is referred to as G. cf. hebetata.

##### Glessula tamakoshi

Taxon classificationAnimaliaGastropodaSubulinidae

Budha & Backeljau sp. n.

http://zoobank.org/1366B4C7-D3B6-4FE5-AFD0-55C7CDD7A400

Figs 2C , 5 , 13A

###### Material examined.

Holotype: CDZMTU057P/1, Suridobhan, left bank of Tamakoshi River, Dolakha District, Central Nepal, North face, rocky hill slope, mixed *Schima wallichi* forest with dominant Lauraceae trees,1023 m, 27.754754N, 86.216755E, 03.II.2009, leg. P.B. Budha. Paratypes: CDZMTU058/13 shells and CDZMTU058P/2 specimens (dissected) from the type locality (same data as holotype).

###### Distribution.

Only known from the type locality.

###### Etymology.

The species name refers to the type locality Tamakoshi River valley.

###### Shell.

Measurements (n = 4): SH 17.7–19.5 mm, SW 9.2–9.6 mm, HA 8.6–8.8 mm, WA 4.9–5.0 mm, Wh 7.0–8.0; holotype: SH 19.5 mm, SW 9.6 mm, HA 8.8 mm, WA 4.9 mm, Wh 7.8. The largest shell measured 19.5 mm, approx. 2× higher than wide, solid, ovate-conic, light yellowish. Surface glossy, with widely spaced incised radial striations, stronger towards the suture and faint at the middle to lower part of the whorls. The first whorl smooth, blunt with fine and dense striations near the suture. Sides convex, suture fairly impressed. Aperture nearly ovate, 1.7× higher than wide, peristome simple and thick, columellar margin abruptly truncate, columella slightly curved.

###### Radula

(Fig. 13A). Teeth pointed, lateral cusps not distinct. Central tooth smaller and with a narrower base than the lateral teeth.

###### Genitalia

**Figure 5.**
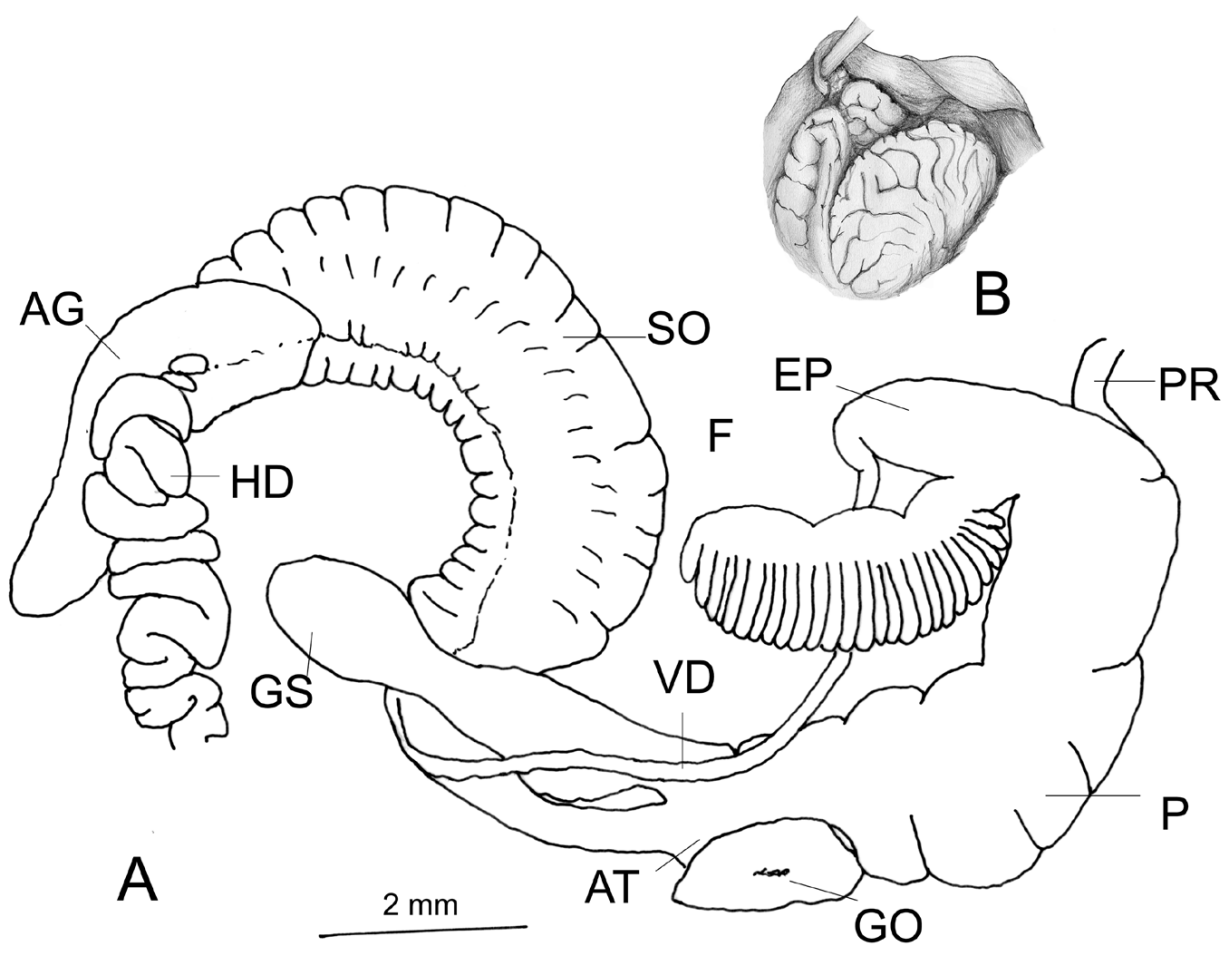
Genitalia of *G. tamakoshi* sp. n., holotype: CDZMTU057P, **A** General view **B** Interior of penis of the same, Suridobhan, left bank of Tamakoshi River, Dolakha District, Central Nepal.

(n = 3) (Fig. 5). Vas deferens with a constant diameter. Flagellum wide comb-like with numerous notches ending in a short hook at the free end. Vagina very short nearly 1/6^th^ length of the penis. Gametolytic sac is oval, connected to the gametolytic duct by a short neck. The penial retractor muscle close to the flagellum. The mass of the hermaphroditic duct highly developed in all dissected samples. Interior of penis with two bulky masses of ‘brain-like folds’ (Fig. 5B).

###### Remarks.

Conchologically, *G. tamakoshi* sp. n. is similar to South Indian *G. indica* Gude, 1914 (genitalia unknown) and Sri Lankan *G. serena* (Benson, 1860). Yet, *G. indica* has a much wider shell and relatively more whorls (>9 whorls), while the penis of *G. serena* contains one longitudinal fold and two folds proximal to this longitudinal fold. Schileyko (1999, p. 542, fig. 711) referred the longitudinal fold to as pilaster and the two proximal folds to as the spiral stimulators. In *G. tamakoshi* sp. n. the interior of the penis contains a bulky masses of brain-like folds (Fig. 5B).

##### Rishetia

Taxon classificationAnimaliaGastropodaSubulinidae

Godwin-Austen, 1920

Ranibania Schileyko and Kuznetsov, 1996, Ruthenica 5: 158–160 (type species: Achatina tenuispira Benson, 1836).

###### Distribution.

India, Sri Lanka, Bangladesh, Myanmar, Nepal (Godwin-Austen 1920, Schileyko 1999, Raheem et al. 2014, Budha et al. 2015).

###### Types species.

*Rishetia longispira* Godwin-Austen, 1920.

###### Main characteristics.

Shell slender, elongately turreted, generally more than 2.5× higher than wide, columella truncated, transluscent, shell sculpture in general stronger than in *Glessula*. Vagina generally longer than penis. The proximal part of the male reproductive organs with a simple flagellum, either like a knob or tubular sac, epiphallic caecum generally present.

##### Rishetia hastula

Taxon classificationAnimaliaGastropodaSubulinidae

(Benson, 1860)

Figs 2D , 6

Achatina hastula B.: Benson 1860, p. 461.
Achatina (Electra) hastula Benson: Hanley and Theobald 1876, pl. 18, fig. 4.
Stenogyra (Glessula) hastula , Benson: Nevill 1878, p. 169.
G. lessula hastula (Benson): Pilsbry 1909, p. 93.
Glessula hastula Benson: Gude 1914, p. 414.
Glessula (Rishetia) hastula Benson: Godwin-Austen 1920, p.16.

###### Material examined.

CDZMTU059/24 shells and CDZMTU059P/5 specimens, Chitwan National Park, Central Nepal, riverine forest, opposite bank of Rapti River at Sauraha, 142–211 m, 27.571774N, 84.489514E, 8.XI.2008. CDZMTU060/6 shells, Kumrose Community Forest, 197 m, 27.556519N, 84.553028E, 21.X.2008. CDZMTU061/1 shell, Baghmara Community Forest, 201 m, 27.57750N, 84.466017E, 20.X.2008, leg. P.B. Budha. *R. hastula* (Benson, 1860) at NHMUK, Godwin-Austen colln. Reg. No. 3557.03.VII.1.

###### Type locality.

West Bengal “Pankabari (= Pankhabari), prope Darjeeling”, India.

###### Distribution.

NE India, Nepal (Budha et al. 2015).

###### Shell.

Measurements (n = 8): SH 9.5–13.6 mm, SW 3.5–4.1 mm, HA 3.3–3.6 mm, WA 2.0–2.5 mm, Wh 8.0–10.0; approx. 3× higher than wide, slender, elongate, brownish, with dense radial riblets all over the shell. The first whorl blunt and smooth, fine riblets starting from the second whorl. Suture deep, spire rounded. Aperture ovate elliptical, nearly 2× higher than wide, peristome thin, columellar margin calloused whitish, obliquely truncate at the base.

###### Genitalia

**Figure 6.**
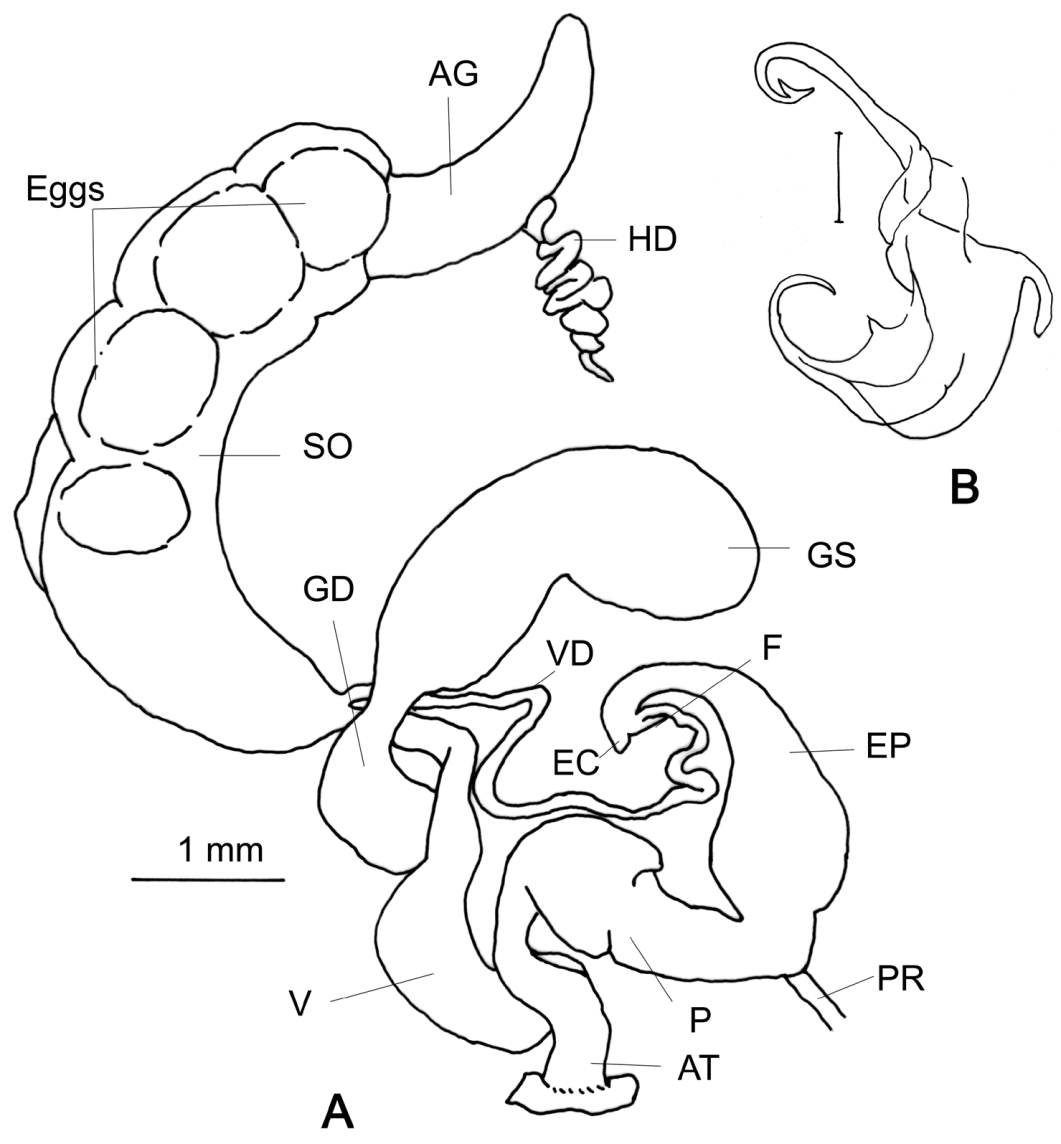
Genitalia of *Rishetia hastula*, CDZMTU059P, **A** General view **B** Spermatophore of the same, Chitwan National Park, riverine forest opposite bank of Sauraha, Rapti River.

(n = 5) (Fig. 6). Flagellum very short tubular sac; there is a very short knob-like epiphallic caecum. Penis cylindrical, basal end comparatively narrow, swollen at the middle and proximal portions cylindrical. Epiphallus basally swollen and the proximal portion tapering. The penial retractor muscle far apart from the flagellum. Gametolytic sac balloon-like, separated from the gametolytic duct by a narrow neck. A mature dissected specimen contained 4–5 gelatinous eggs in the spermoviduct. Another mature specimen contained a spermatophore in the gametolytic sac. This is the first observation of a glessuline spermatophore (Fig. 6B). Vagina short, nearly half the length of the penis. Albumen gland elongate and yellowish; hermaphroditic duct thinner than the albumen gland in the observed specimens.

###### Remarks.

*R. hastula* is common in subtropical riverine floodplain forest leaf litter at lower altitudes (up to 300 m) in Chitwan National Park and the adjacent bufferzone community forests.

##### Rishetia kathmandica

Taxon classificationAnimaliaGastropodaSubulinidae

Budha & Backeljau sp. n.

http://zoobank.org/44995A5D-D1BC-4B7F-ADC8-B64DBE3015E9

Figs 2E , 7 , 13B

Ranibania tenuispira (Benson, 1836): Schileyko and Kuznetsov 1996, p. 158.
Rishetia tenuispira (Benson, 1836): Schileyko 1999, p. 533.
Rishetia tenuispira (Benson, 1836): Budha et al. 2015, p. 17.

###### Material examined.

Holotype. CDZMTU062P/1 specimen (dissected), Godawari Botanical Garden, Lalitpur, 1453–1550 m, 27.600013N, 85.398443E, 30.IV.2007, leg. P.B. Budha and R. Devkota. Paratypes: CDZMTU62/20 shells and CDZMTU063P/2 specimens (one dissected) from the type locality (same data as holotype). Paratypes: CDZMTU062b/40 shells and CDZMTU062P/7 specimens, Godawari Botanical Garden, Lalitpur, 1453 m, 27.596657N, 85.381392E, 03.IX.2008, leg. P.B. Budha. Paratypes: CDZMTU064/40 shells and CDZMTU064P/12 specimens (3 dissected), Nagarjun Forest, Balaju, Kathmandu, 1600 m-1800 m, 27.739058N, 85.297854E, 02.X.2008, leg. S. Khatiwara and S. Khanal. *Glessula* sp. Nagarkot, Nepal, A. Comfort 1989, one shell at NHM, London (general collection, non-type). *Rishetia tenuispira* (Benson, 1836). In addition, one photograph of two shells (Fig. 14D): ZMMU, Lc. 34221, Raniban Range, Nagarjun Royal Forest, Balaju, Kathmandu, Nepal, 1480 m, leg./det. A.G. Kuznetsov, 28.04.1995; ZMMU, Lc. 34222, 1600–1800 m, leg./det. A.G. Kuznetsov, 28+30.04.1996.

###### Distribution.

Nepal.

###### Etymology.

The name refers to the hill forests of Kathmandu valley from where the specimens were collected.

###### Shell.

Measurements (n = 17): SH 24.8–41.1 mm, SW 7.8–9.3 mm, HA 7.1–8.5 mm, WA 4.1–5.4 mm, Wh 11.0–13.0; holotype: SH 28.1 mm, SW 8.1 mm, HA 7.1 mm, WA 4.1 mm, Wh 12; approx. 4× higher than wide, elongately turreted, colour ruddy, regularly sculptured. The first whorl pointed (Fig. 2E1) with distinct and regular radial riblets, more prominent on the first few whorls, ribs much stronger towards the suture; middle whorls of the shell show incised radial striation. Suture shallow, sides moderately flat. Aperture small, oval, 1.6× higher than wide, peristome thin, columellar margin slightly convex, thinly calloused, white.

###### Radula

(Fig. 13B). Central tooth very small, lacks cusps, lateral teeth tricuspid.

###### Animal

(Fig. 14E). Grey black, with dark tentacles. Body minutely papillate. The sole is lighter than the body.

###### Genitalia

**Figure 7.**
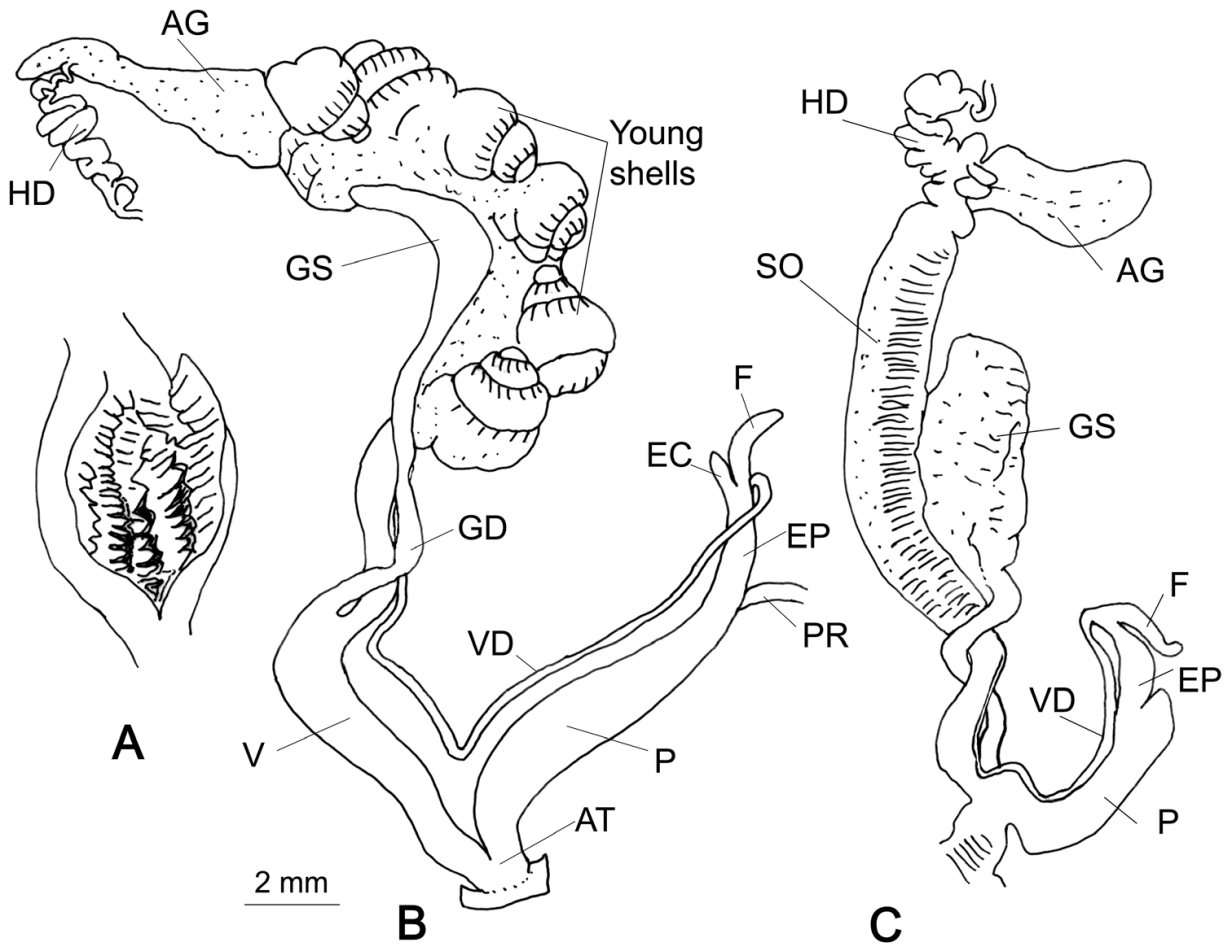
Genitalia of *R. kathmandica* sp. n. and Glessula (Rishetia) longispira Godwin-Austen, 1920. **A** Interior of penis of *R. kathmandica* sp. n., holotype CDZMTU062P **B** General view of the same, Godawari Botanical Garden, Lalitpur, Central Nepal **C** General view of *G.* (*R.*) *longispira* reproduced from Godwin-Austen (1920), pl. CLXV, fig. 4.

(n = 5) (Fig. 7A–B). The flagellum is a long cylindrical sac; the epiphallic caecum is a simple sac, shorter than the flagellum. Penis cylindrical and longer than the epiphallus. The penial retractor muscle far apart from the flagellum. Interior wall of the penis with distinct rectangular zigzag folds (Fig. 7A). Three specimens contained 12–14 juvenile shells in the spermoviduct. Vagina about as long as the penis. Gametolytic sac pear shaped with a neck that is not distinct from the Gametolytic duct. Hermaphroditic duct is connected closer towards the free end of the albumen gland.

###### Remarks.

Schileyko and Kuznetsov (1996) initially described this species as *Ranibania tenuispira* (Benson, 1836) (collected from Raniban forest, Balaju, Kathmandu, Nepal). Later Schileyko (1999) referred the species to as *Rishetia tenuispira* (Benson, 1836). However, the shells of Nepalese “*R. tenuispira*” do not match with: (1) the possible syntype of *Subulina tenuispira* Benson, 1836 at UMZC l.102045, type locality labelled as ‘Teria Ghat’, because this syntype has a blunt first whorl (Fig. 14A), (2) *Achatina tenuispira* Benson, 1836 shells in the MacAndrew collection from the W. Khasi hills (India) at the NHMUK, London, Acc. No. 1582.03.VII.I (Fig. 14C), and (3) 33 syntypes of Glessula (Rishetia) longispira Godwin-Austen, 1920 from Rishetchu, Sikkim, India NHMUK, Reg. No. 1903.7.1.552, because these syntypes have a blunt first whorl and comparatively very slender shell (Fig. 14B). Therefore we here describe Nepalese “*R. tenuispira*” as the new species *R. kathmandica*. The genitalia of this new species are similar to those of *R. longispira*, but *R. kathmandica* sp. n. has a relatively longer vagina (Fig. 7B, C). *R. kathmandica* sp. n. is common in mixed *Quercus-Rhododendron* forests between 1400 m and 2000 m in the hills around Kathmandu valley. The general collection of NHM, London contains a single shell of *R. kathmandica* sp. n. from Nagarkot, Nepal, labelled as *Glessula* sp.

##### Rishetia cf. mastersi

Taxon classificationAnimaliaGastropodaSubulinidae

Godwin-Austen, 1920

Figs 2F , 8A

Glessula (Rishetia) mastersi Godwin-Austen, 1920: Godwin-Austen 1920, p. 46, pl. 161, fig. 14., pl. 162, fig. 22.
Glessula mastersi Godwin-Austen, 1920: Ramakrishna et al. 2010, p. 166.

###### Material examined.

CDZMTU065/5 shells, Kurintar, Chitwan, degraded riverine bushes with big boulders, mixed *Shorea robusta* forest, 420–527 m, 27.875820N, 84.589321E, 25.X.2008, leg. P.B. Budha. CDZMTU065b/16 shells and CDZMTU065P/1 specimen (dissected), Kurintar, Chitwan, 527 m, 27.874143N, 84.586683, 23.VII.2010, leg. P.B.Budha.

###### Shell.

Measurements (n = 30): SH 13.0–19.0 mm, SW 5.4–6.3 mm, HA 5.0–6.1 mm, WA 2.7–3.5 mm, Wh 8.5–9.5; approx. 2.7× higher than wide, oblong turreted, yellowish brown. Surface with shallow radial ribs, stronger towards the suture, sculpture regular, thin transparent periostracum. Suture deep, spire convex. The first whorl rounded (Fig. 2F1). Aperture small and ovate, 1.7× higher than wide, peristome thin, columellar margin short and abruptly truncated.

###### Genitalia

**Figure 8.**
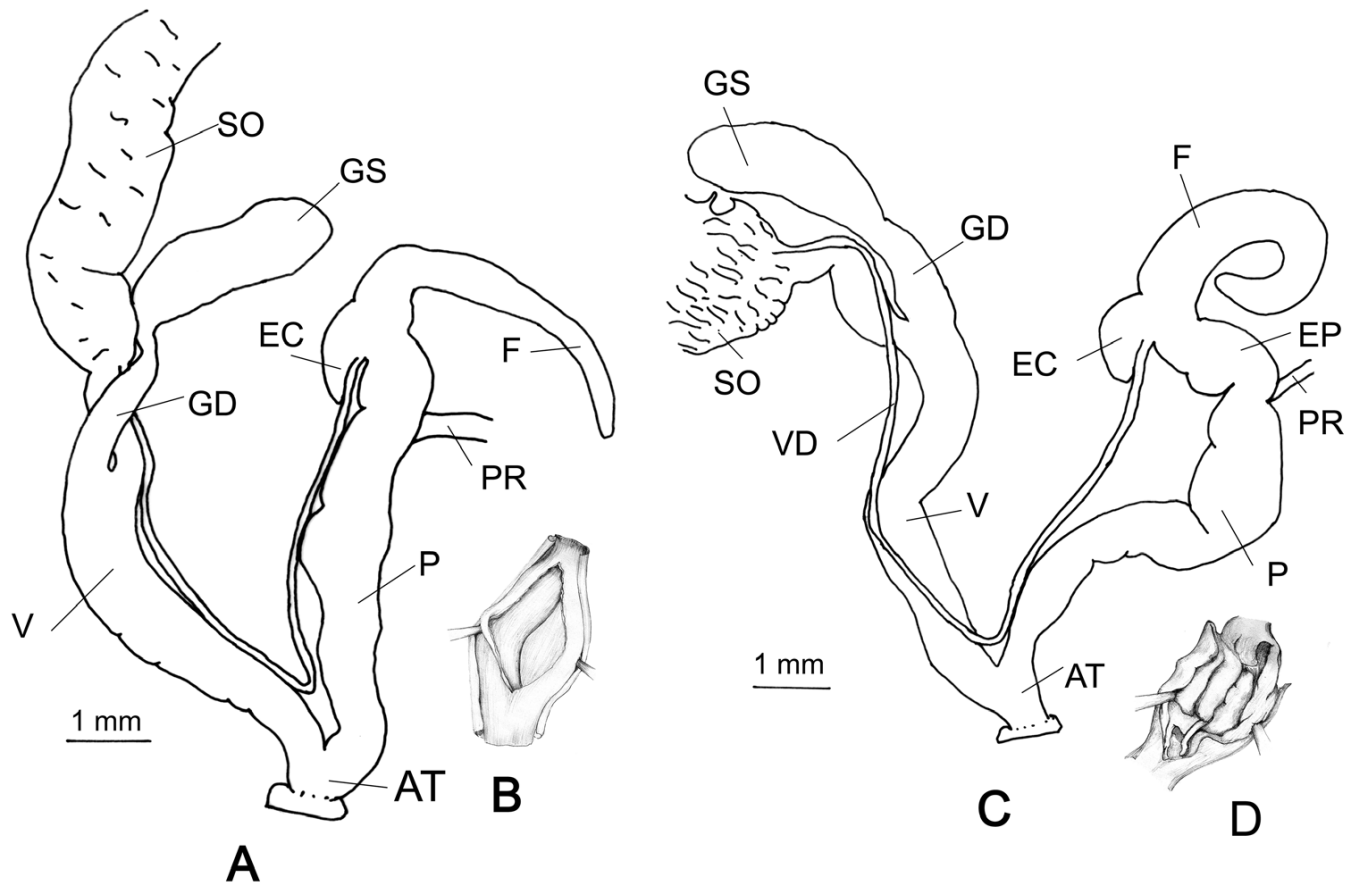
Genitalia of R. cf. mastersi and *Rishetia* sp.: **A** General view of R. cf. mastersi, CDZMTU065P **B** Interior of penis of the same, Kurintar, Chitwan, degraded riverine bushes with big boulders, mixed *Shorea robusta* forest **C** General view of *Rishetia* sp. CDZMTU078P **D** Interior of penis of the same, Boshikharka, Dhading, Central Nepal.

(n = 2) (Figs 8A). Vas deferens long with a constant diameter. The flagellum is a simple sac. Epiphallic caecum is longer than the flagellum. Epiphallus much shorter than the penis. The penial retractor muscle far apart from the flagellum. Gametolytic duct slender, ending into a balloon-like gametolytic sac, which is slightly longer than the gametolytic duct. Vagina as long as the penis. Interior wall of the penis with a strong fold.

###### Remarks.

A single specimen of *Rishetia* sp. was collected from the *Shorea robusta* forest at Bosikharka, Dhanding (CDZMTU078P). Its shell is similar to *R. mastersi* Godwin-Austen, 1920, but it is slightly smaller and has relatively stronger radial ribs. The shape of its flagellum and epiphallus is similar to that of *R. mastersi* too (Figs 8A, 8C), but the interior of its penis shows three longitudinal folds (Fig. 8D), whereas in *R. mastersi* there is only one longitudinal fold (Fig. 8B).

##### Rishetia nagarjunensis

Taxon classificationAnimaliaGastropodaSubulinidae

Budha & Naggs sp. n.

http://zoobank.org/CB41BEB9-DCC9-47F5-9E13-7AC9A357BDDF

Figs 2G , 9 , 13E

###### Material examined.

Holotype: CDZMTU067P/1, Nagarjun Forest, Balaju-Jamacho trail, Nagarjun-Shivapuri National Park, Kathmandu, Nepal, 1850 m, 27.745997N, 85.287240E, 24.I.2009, leg. P.B. Budha, R. Devkota, S. Khatiwara and S. Khanal. Paratypes: CDZMTU067/9 shells and CDZMTU068P/1 specimen from the type locality (same data as holotype). Paratypes: CDZMTU069P/1 specimen (dissected), Phulchowki Hill, mixed *Rhododendron* forest, 2324 m, 27.578317N, 85.396885E, 03.V.2007, leg. P.B. Budha.

###### Distribution.

Nagarjun-Shivapuri National Park and Phulchowki Hill, Central Nepal.

###### Etymology.

The name is derived from the type locality Nagarjun Forest.

###### Shell.

Measurements (n = 4): SH 33.2–38.4 mm, SW 10.6–11.9 mm, HA 10.2–10.8 mm, WA 5.5–6.2 mm, Wh 10–11.0; holotype: SH 38.0 mm, SW 12.0 mm, HA 10.5 mm, WA 6.3 mm, Wh 11; approx. 3.3× higher than wide, elongately turreted, thick, reddish-brown or dark chestnut colour. Surface striated with obliquely raised riblets on the first 2–3 whorls, later whorls with comparatively thin and dense sculpture. The first whorl smooth, rounded. Sides rather flat, suture shallow. Aperture ovate, 1.8× longer than wide, peristome thin, inner side of the aperture thickened and whitish, columella slightly truncate at the base.

###### Radula

(Fig. 13E). Central tooth very small, lacks cusps, lateral teeth shouldered.

###### Genitalia

**Figure 9.**
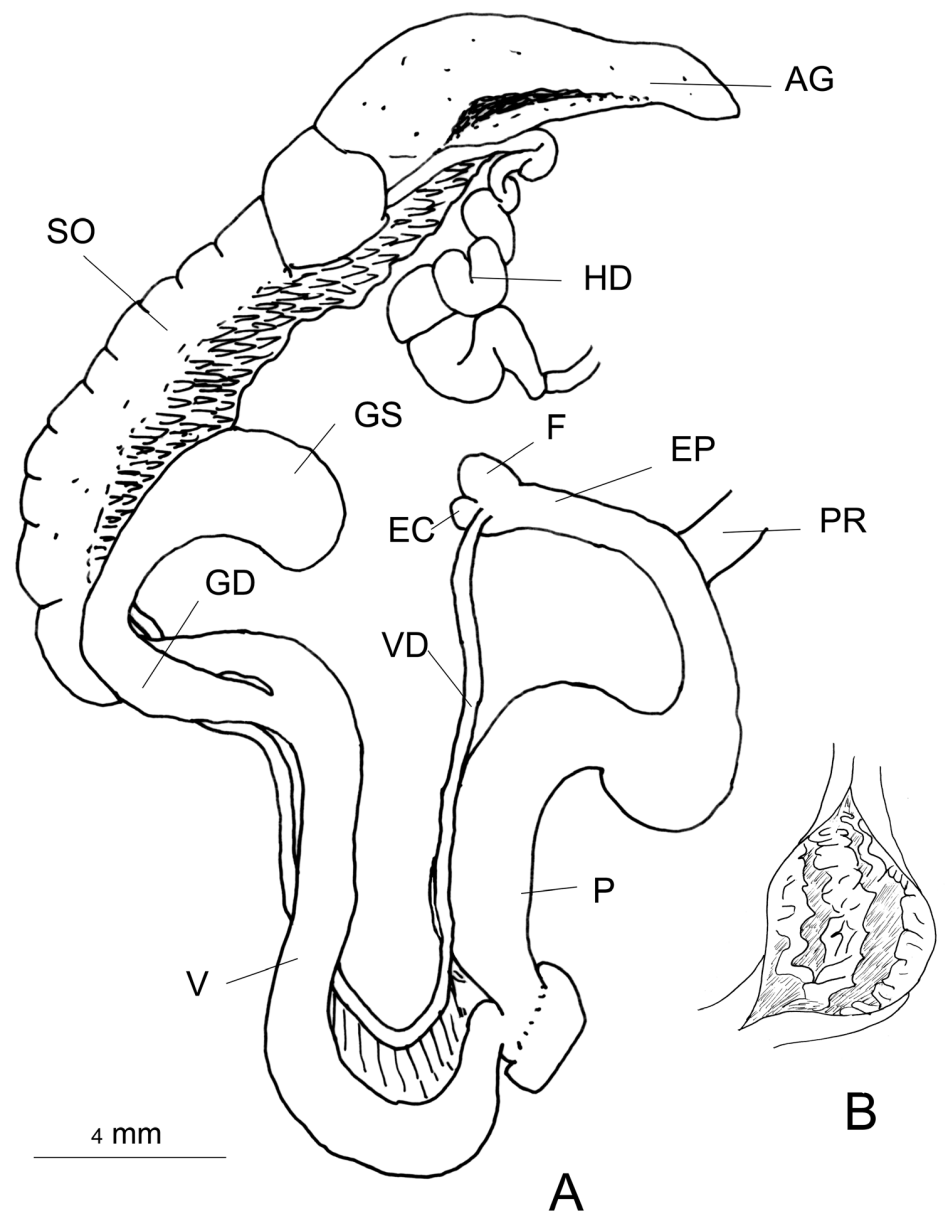
Genitalia of *R. nagarjunensis* sp. n., holotype: CDZMTU067P, **A** General view **B** Interior of penis of the same, Nagarjun forest, Balaju-Jamacho trail Nagarjun-Shivapuri National Park, Kathmandu, Nepal.

(n = 2) (Fig. 9). All specimens have a long vas deferens. Flagellum and epiphallic caecum are reduced to two very short knobs (Fig. 9A). Penis cylindrical, with a nearly constant diameter in its proximal half, distally it rapidly expands into a bulbous section before the diameter contracts to somewhat less than the proximal penis, narrowing slightly distally. The penial retractor muscle far apart from the flagellum. The inner surface contains weakly convoluted folds (Fig. 9B). Epiphallus length about 1/3 of penis length. Vagina nearly as long as the penis. Gametolytic duct cylindrical, ends into a simple gametolytic sac. Hermaphroditic duct is loosely convoluted and connected at the middle of the albumen gland.

###### Remarks.

The shell of *R. nagarjunensis* sp. n. is similar in size and shape to that of sympatric *R. kathmandica* sp. n. However, the shell of *R. kathmandica* sp. n. is more slender and has more whorls than *R. nagarjunensis* sp. n. Conversely, *R. nagarjunensis* sp. n. has a wider body whorl, a comparatively more robust shell, and stronger radial ribs than *R. kathmandica* sp. n. The genitalia of both species are consistently different due to the reduced knob-like flagellum and epiphalic caecum in *R. nagarjunensis* sp. n., as well as by the inner surface of the penis, which in *R. nagarjunensis* sp. n. shows three loosely convoluted folds, whereas in *R. kathmandica* sp. n. it shows distinct rectangular zigzag folds (Fig. 7A1).

##### Rishetia rishikeshi

Taxon classificationAnimaliaGastropodaSubulinidae

Budha & Naggs sp. n.

http://zoobank.org/86CA7567-2A6B-42AD-B7C6-6DEAB188F935

Figs 2I , 10 , 13C

###### Material examined.

Holotype: CDZMTU070P/1 specimen, Jhawalepakho Community Forest near Rishikesh Temple, Ridi, Gulmi District, montane hill *Shorea robusta* forest, 832 m, 27.932775N, 83.436552E, 06.IX.2006, leg. P.B. Budha. Paratypes: CDZMTU071/11 shells from the type locality (same data as holotype).

###### Distribution.

Only reported from the type locality.

###### Etymology.

The species name refers to the famous Rishikesh Hindu Temple at Ridi, Gulmi District.

###### Shell.

Measurements (n = 10): SH 12.1–16.2 mm; SW 4.6–5.7 mm; HA 4.0–5.5 mm; WA 2.5–3.4 mm; Wh 8.0–9.3; holotype: SH 14.1 mm; SW 5.3 mm; HA 4.4 mm; WA 2.6 mm; Wh: 9.2; approx. 2.6× higher than wide, oblong turreted, yellowish brown. Surface with regular, dense, radial ribs towards the suture. Suture deep, spire convex. Aperture small and ovate, 1.7× higher than wide, peristome thin, columellar margin abruptly truncated.

###### Radula

(Fig. 13C). Central tooth very small, lateral teeth tricuspid with the central cusp bifurcated.

###### Animal

(Fig. 14E). Dark grey with a heavily papillated body. The foot is light, showing weak transverse lines on the sole.

###### Genitalia

**Figure 10.**
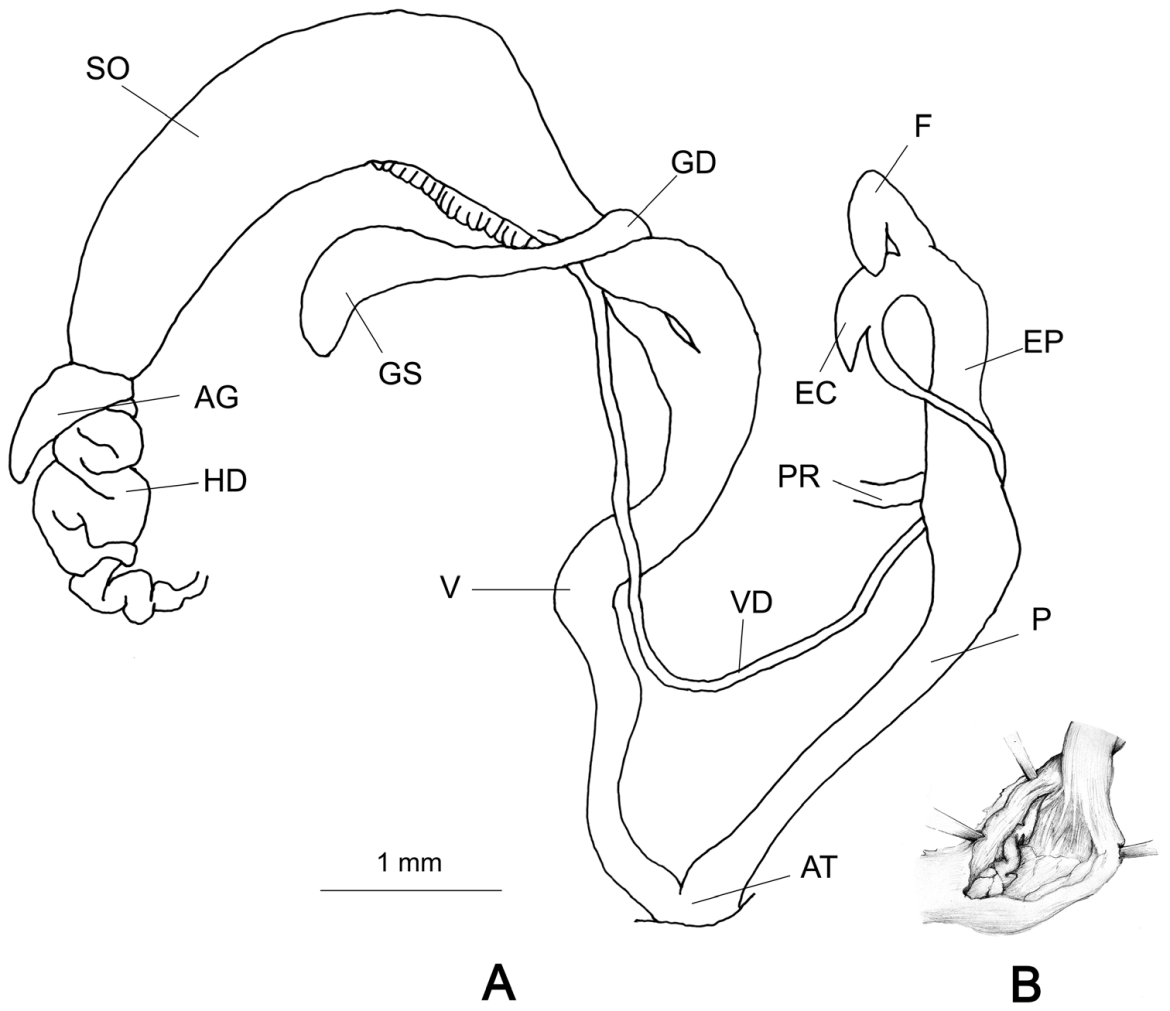
Genitalia of *R. rishikeshi* sp. n., holotype: CDZMTU0170P, **A** General view **B** Interior of penis of the same, Jhawalepakho Community Forest, Ridi, Gulmi District, montane hill *Shorea robusta* forest.

(n = 2) (Figs 10A–B). Vas deferens with a constant diameter over its entire length. Epiphallic caecum nearly as long as the flagellum. Epiphallus long, but shorter than the penis. The penial retractor muscle far apart from the flagellum. Vagina as long as the penis. Gametolytic sac is nearly round, separated by a neck from the gametolytic duct. Albumen gland very small in the dissected specimens. The internal surface of the penis smooth with several longitudinal convoluted folds (Fig. 10B).

###### Remarks.

*R. rishikeshi* sp. n. was collected from the western side of Kaligandaki River. Conchologically, this new species is similar to *R. mastersi* Godwin-Austen, 1920 of Assam, NE India (Godwin-Austen 1920, p. 46, pl. 162, fig. 3). But the flagellum and epiphallic caecum are comparatively very short in *R. rishikeshi* sp. n. Similarly the interior of the penis in *R. rishikeshi* sp. n. contains several convoluted folds while in *R. mastersi* there is only one strong and straight fold.

##### Rishetia subulata

Taxon classificationAnimaliaGastropodaSubulinidae

Budha & Naggs sp. n.

http://zoobank.org/F44A7F59-8DB7-4715-B55A-D6D9D12B1691

Figs 2J , 11

###### Material examined.

Holotype: CDZMTU072P/1 specimen (dissected), Godawari, along the Godawari-Phulchowki road, approx. 200 m above the Naudhara Temple, 1837 m, 27.5766N, 85.3786E, 02.X.2008, leg. P.B. Budha. Paratype: CDZMTU072/1 shell (same locality). Paratypes: CDZMTU073/3 shells, Phulchowki Hill, Central Nepal, mixed forest, 2324 m, 27.578317N, 85.396885E, 04.V.2007, leg. P.B. Budha and R. Devkota.

###### Distribution.

Only known from the type locality.

###### Etymology.

The name refers to the typical subuline-like shell shape.

###### Shell.

Measurements (n = 3): SH 10.8–14.9 mm, SW 3.6–4.1 mm, HA 3.3–3.5 mm, WA 1.8–1.9 mm, Wh 9.0–9.5; holotype: SH 10.8 mm, SW 3.6 mm, HA 3.3 mm, WA 1.8 mm, Wh 9.0; approx. 3.1× higher than wide, elongated, thin, dull brown. Surface shining obliquely striated, covered with thin transparent epidermis, slightly denser on the 3^rd^ and 4^th^ whorl; transverse sculpture more widely separated on the penultimate and body whorls. Whorls shouldered. The first whorl blunt and smooth, eroded. Sides rounded; suture deep. Aperture ovate elliptical, approx. 2× higher than wide, peristome thin, columellar margin calloused whitish, slightly truncate at the base.

###### Radula

(Fig. 13D). Central tooth very small, lacks cusps, lateral teeth tricuspid.

###### Animal.

Dark grey and papillate.

###### Genitalia

**Figure 11.**
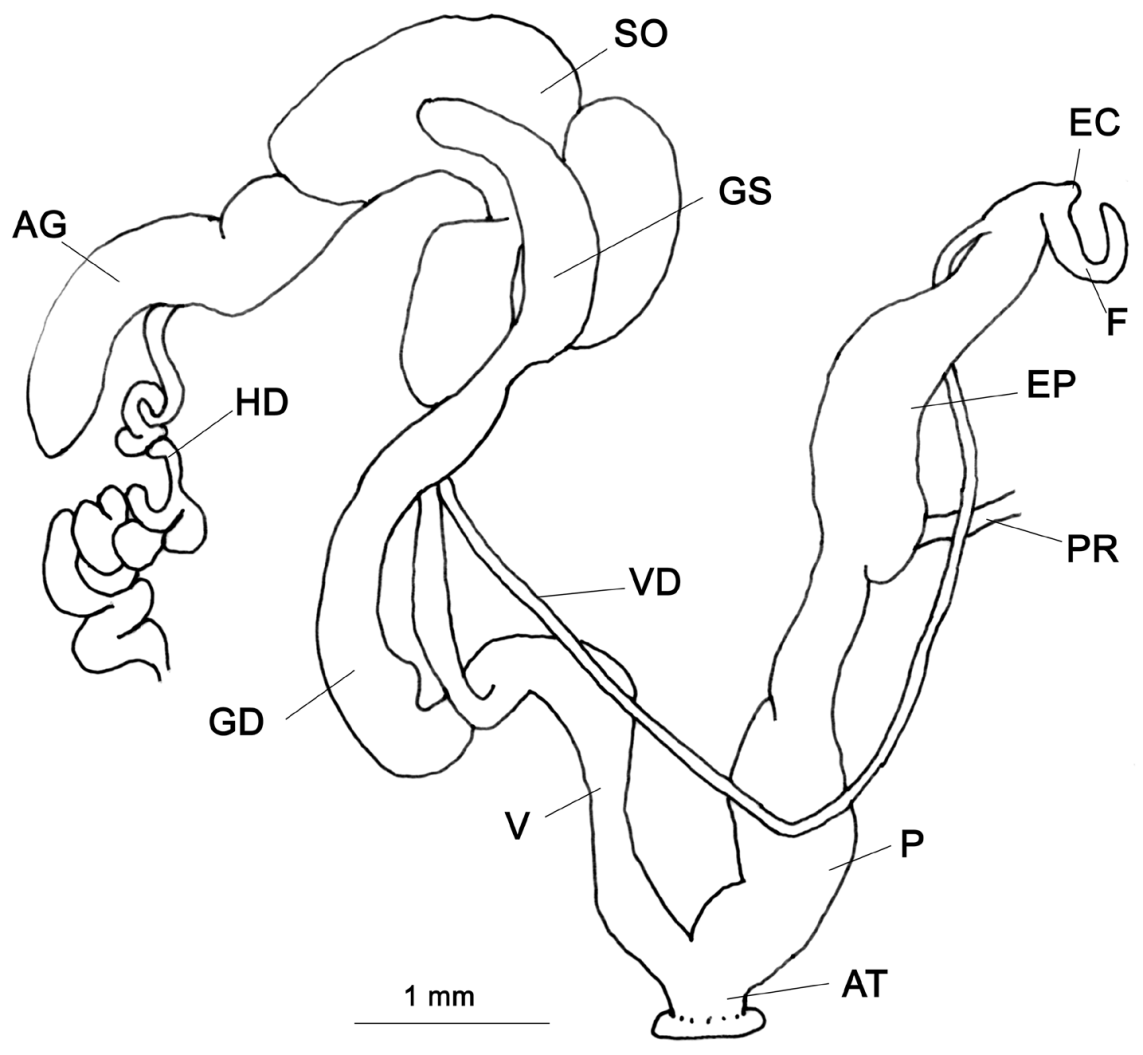
Genitalia of *R. subulata* sp. n., holotype: CDZMTU072P, Godawari, along the Godawari-Phulchowki road approx. 200 m above the Naudhara Temple.

(n = 1) (Fig. 11). Vas deferens very long, enters into the epiphallus at some distance from the base of the flagellum. The flagellum is long and C-shaped. Epiphallic caecum very short. Epiphallus approx. as long as the penis. The basal part of the penis is comparatively wider than the proximal part. The penial retractor muscle far apart from the flagellum. Gametolytic sac elongated. The vagina is nearly as long as the penis. Albumen gland elongated and hermaphrodite duct is connected at the middle of the gland.

###### Remarks.

*R. subulata* sp. n. is similar to *R. hastula* (Benson, 1860) and *R. tribhuvana* sp. n., but *R. subulata* sp. n. has a wider body whorl and more blunt first whorl than *R. hastula*, while its 2^nd^ and 3^rd^ whorls have equal diameters (unequal diameters in *R. hastula*) (Fig. 2 D1 and J1). The body whorl in *R. subulata* sp. n. is much wider than in *R. tribhuvana* sp. n. (Fig. 2 J and K). Moreover, the flagellum of *R. subulata* sp. n. much larger than the epiphallic caecum, whereas in *R. tribhuvana* sp. n. the flagellum and epiphallic caecum are of similar sizes. In *R. hastula* the flagellum and the epiphallic caecum are very small.

##### Rishetia tribhuvana

Taxon classificationAnimaliaGastropodaSubulinidae

Budha sp. n.

http://zoobank.org/F9E43F89-6DAA-4849-9447-2D5F9C91EA6D

Figs 2K , 12

###### Material examined.

Holotype: CDZMTU074P/1 specimen, Tribhuvan University garden, Kirtipur, Kathmandu, Nepal, 1320 m, 27.680203N, 85.289154E, 15.VIII.2006, leg. P.B. Budha. Paratypes: CDZMTU074/15 shells and CDZMTU075P/5 specimens (3 dissected) from the type locality, 22.III.2011, leg. P.B. Budha. Paratypes: CDZMTU076/6 shells, Champadevi Forest, Kirtipur, Kathmandu, Nepal, 1680 m, 27.653060N, 85.244785E, 23.VIII.2006, leg. P.B. Budha. Paratypes: CDZMTU077/4 shells, Nagarjun Forest, 1582 m and 1680 m, 27.742616N, 85.293248E, 07.I.2009, 16.I.2009, leg. S. Khanal and S. Khatiwara.

###### Distribution.

Hill forests of Kathmandu valley, Nepal.

###### Etymology.

The name refers to the type locality, Tribhuvan University garden

###### Shell.

Measurements (n = 12): SH 7.5–11.0 mm, SW 2.7–3.0 mm, HA 2.5–3.0 mm, WA 1.4–2.0 mm, Wh 8.5–9.5; holotype: SH 10.1 mm, SW 3.0 mm, HA 2.5 mm, WA 2.0 mm, Wh 9; approx. 3.3× higher than wide, slender, thin, dull brown. Surface striated with radial fine ribs, much denser on the 3^rd^ and 4^th^ whorls; ribs widely separated on the penultimate and body whorls. The first whorl blunt and smooth. Spire rounded; suture deep. Aperture ovate elliptical; approx. 2× higher than wide, peristome thin; columellar margin calloused whitish, obliquely truncate at the base.

###### Genitalia

**Figure 12.**
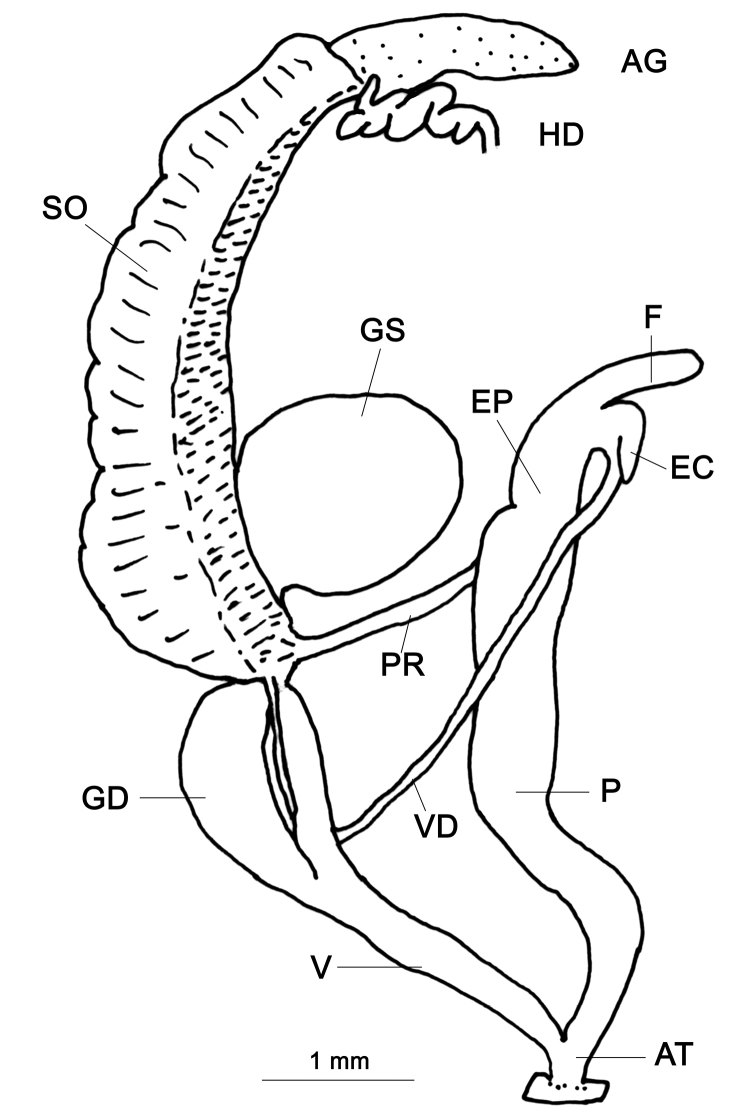
Genitalia of *R. tribhuvana* sp. n., holotype: CDZMTU077P, Tribhuvan University garden, Kirtipur, Kathmandu, Nepal.

(n = 4) (Fig. 12). Vas deferens long. Flagellum slightly shorter than the epiphallic caecum. The length of the flagellum nearly two-thirds of that of the epiphallus. The basal part of the penis narrower than the proximal part. The vagina nearly as long as the penis. The penial retractor muscle far apart from the flagellum. The gametolytic sac is rounded and distinct from the gametolytic duct. The hermaphrodite duct is connected at the base of the albumen gland.

###### Remarks.

*R. tribhuvana* sp. n. is the smallest *Rishetia* species in Nepal. Conchologically, it is “intermediate” between *R. hastula* (Benson, 1860) and *R. roberti* Godwin-Austen, 1920. *R. tribhuvana* sp. n. differs from both these species by its weaker radial sculpture compared to *R. hastula* and its narrower body whorl compared to *R. roberti*. Moreover, the illustrations of Godwin-Austen (1920, pl. CLXIII, fig. 10) suggest that *R. roberti* has a rounded first whorl, whereas it is nearly flat in *R. tribhuvana* sp. n. The flagellum and epiphallic caecum are well-developed and nearly equal in size in *R. tribhuvana* sp. n., whereas in *R. hastula* the flagellum is very small and the epiphallic caecum is only a minute-knob. *R. tribhuvana* sp. n. and *R. hastula* are known from geographically different locations. The former is a hill species reported above 1300 m, while the latter is known only from the plain below 300 m elevation. *R. roberti* is also a hill species from Richila peak, Sikkim. The presence of *R. tribhuvana* sp. n. in the garden of Tribhuvan University is due to a historical connection between this garden and the Champadevi hill Forest, about 4 km south 300–400 m higher from the university premises. This connection has been lost because of human settlement.

**Figure 13.**
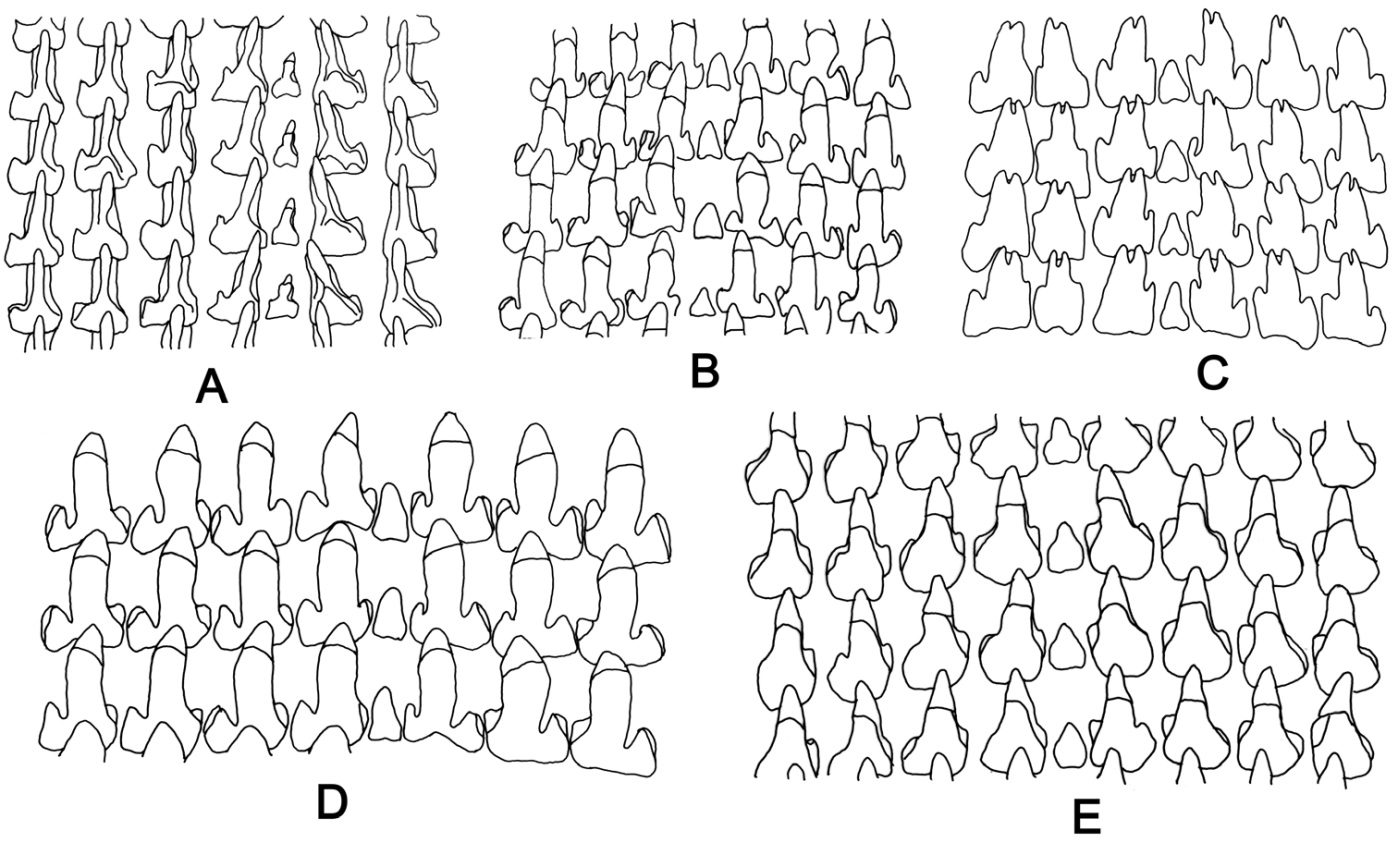
Radula of *Glessula* and *Rishetia*. **A** *G. tamakoshi* sp. n., CDZMTU057P, Suridobhan, Dolakha **B** *R. kathmandica* sp. n., CDZMTU062P, Godawari, Lalitpur **C** *R. rishikeshi* sp. n., CDZMTUO70P, Jhawalepakho, Ridi, Gulmi **D** *R. subulata* sp. n., CDZMTU072P, Godawari, Lalitpur **E** *R. nagarjunensis* sp. n., CDZMTU067P, Raniban, Balaju, Kathmandu.

**Figure 14.**
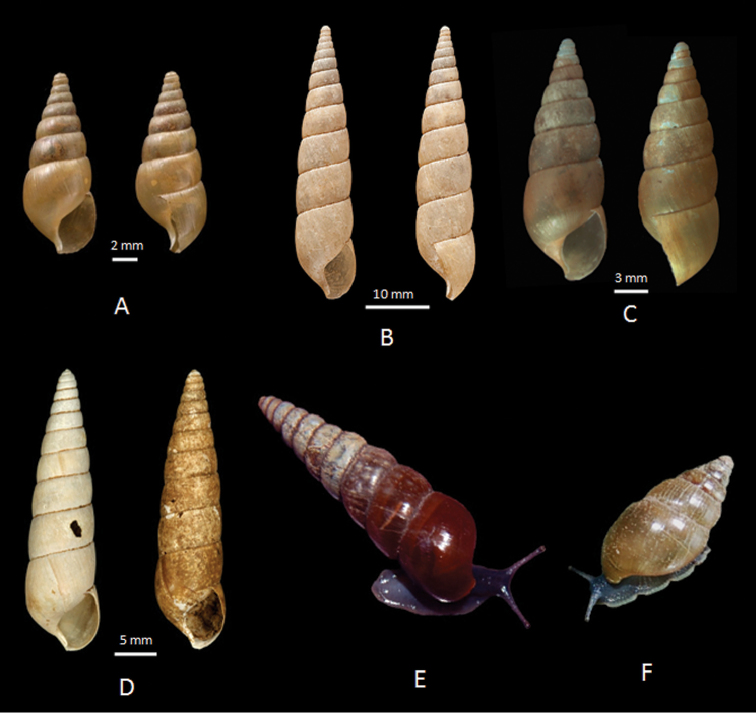
Shells and animals of glessulines. **A** *Achatina tenuispira*, possible syntype from ‘Teria Ghat’ labelled as *Subulina tenuispira* Bens. ZMCU l.102045, Cambridge **B** *Rishetia longispira* Godwin-Austen, 1920, 33 syntypes, Rishetchu, Sikkim, NHMUK, Reg. No. 1903.7.1.552 **C** *Rishetia tenuispira*, W. Khasi Hills NHMUK, London, Acc. No. 1582.03.VII.I **D** Two shells of *Rishetia kathmandica* sp. n. from Nepal, previously determined by Schileyko (1999) as *Rishetia tenuispira* (Benson, 1836) (two lots : Central Nepal, Kathmandu valley, 1.3 km NW from Balaju, Rani-Ban Range, Nagarjun Royal Forest, 1480 m a.s.l., leg./det. A.G. Kuznetsov, 28.04.1995. ZMMU, No. Lc-34221 and 1600–1800 m a.s.l., leg./det. A.G. Kuznetsov, 28+30.04.1996. ZMMU, No. Lc-34222 **E** *Rishetia kathmandica* sp. n. Godawari, Lalitpur **F** *Rishetia mastersi*, Kurintar, Nepal.

#### Key to the Nepalese Glessulinae

**Table:** 

1	Shell ovate-conic, adult shell height/width ratio < 2.5	**2** (*Glessula*)
–	Shell elongately turreted, adult shell height/width ratio > 2.5	**4** (*Rishetia*)
2(1)	First two whorls with spiral lirae (Fig. 2A1), flagellum hand-shaped (Fig. 3)	***G. orobia***
–	First two whorls without spiral lirae, flagellum comb-shaped (Figs 4–5)	**3**
3(2)	Adult shell height < 15 mm, flagellum comb-shaped, vas deferens becomes wider towards spermoviduct (Fig. 4)	***G. hebetata***
–	Adult shell height > 15 mm, flagellum comb-shaped, diameter of vas deferens constant (Fig. 5)	***G. tamakoshi* sp. n.**
4(1)	Adult shell height < 20 mm	**5**
–	Adult shell height > 20 mm	**9**
5(1)	Adult shell height/width ratio ≤ 3	**6**
–	Adult shell height/width ratio > 3	**8**
6(5)	Flagellum long	**7**
–	Flagellum very short, epiphallic caecum very short-knob (Fig. 6)	***R. hastula***
7(5)	Epiphallic caecum longer than flagellum (Fig. 8)	***R. mastersi***
–	Epiphallic caecum nearly as long as flagellum (Fig. 10)	***R. rishikeshi* sp. n.**
8(5)	Epiphallic caecum much shorter than flagellum (Fig. 11)	***R. subulata* sp. n.**
–	Epiphallic caecum nearly as long as flagellum (Fig. 12)	***R. tribhuvana* sp. n.**
9(4)	Shell slender, 10–14 whorls, flagellum and epiphallic caecum simple sacs (Fig. 7)	***R. kathmandica* sp. n.**
–	Shell slender, 10–11 whorls, flagellum and epiphallic caecum simple knob (Fig. 9)	***R. nagarjunensis* sp. n.**

## Discussion

Godwin-Austen (1920) differentiated *Rishetia* from *Glessula* *sensu stricto* on the basis of the male reproductive organs of four *Glessula* species *G. ochracea*, G. orobia var. major, *G. orophila* and *G. inornata*, and two species of *Rishetia*, *R. longispira* (type species) and *R. garoense* Godwin-Austen, 1920. He reported that *Glessula* has a comb-like flagellum and *Rishetia* has a simple sac-like flagellum. Probably based on the elongated shell of *R. longispira* he assigned several other slender and elongated Himalayan species to *Rishetia*. Therefore we explored the relation between shell form and genitalia in 10 species of *Glessula* and 10 species of *Rishetia* (Table 1). This suggests that the male reproductive organ of *Glessula* and *Rishetia* differs by: (1) the shape of the flagellum, being hand- or comb-shaped without an epiphallic caecum in *Glessula* (Figs 3–5) vs. a simple knob or tubular sac with an epiphallic caecum in *Rishetia* (Figs 6–12), (2) the penial retractor inserting close to the flagellum and epiphallus in *Glessula* vs. penial retractor inserting far from the flagellum near the penis/epiphallus junction in *Rishetia*, and (3) the very short epiphallus in *Glessula* vs. the comparatively longer epiphallus in *Rishetia*. Recent data on the genitalia of *G. ceylanica* (the type species of *Glessula*) shows that this species has a very distinct hand-like flagellum with a small thumb and five fingers (D. Raheem, pers communication). Taken altogether, it appears as if these genital characters are correlated with the shell height/width ratio, such that the shell height/width ratio in *Glessula* is < 2.5, while in *Rishetia* it is always > 2.5, at least in the species listed in Table 1. Also the shell sculpture tends to differ between *Glessula* and *Rishetia*, with *Glessula* usually having a somewhat weaker sculpture than *Rishetia*. The extent to which this putative differentiation between *Glessula* and *Rishetia* can be maintained when data on more species from a wider geographic range become available remains to be established.

**Table 1.** Qualitative correlation between shell height/width ratio and proximal part of the male genital parts in *Glessula* and *Rishetia* (n refers to the number of shells measured).

Species	SH	SW	SH/SW ratio	Flagellum, epiphallic caecum (EC)	Reference
*Glessula ceylanica* (n=2)	23.0	11.0	2.1	Hand-shaped, EC absent	4, 1
G. cf. hebetata (n=1)	13.3	6.2	2.1	Comb-shaped, EC absent	1
*G. inornata* (n=3)	27.0	12.0	2.2	Comb-shaped, EC absent	2, 7
*G. oakesi* (n=2)	13.8	6.0	2.3	Hand-shaped, EC absent	2
*G. ochracea* (n=1)	21.3	9.3	2.3	Comb-shaped, EC absent	2,3
*G. orobia* (n=6)	7.7	4.3	1.8	Hand-shaped, EC absent	1
G. orobia var. major (n=2)	11.8	5.8	2.0	Hand-shaped, EC absent	2
*G. orophila* (n=4)	19.2	9.6	2.0	Comb-shaped, EC absent	2
*G. serena* (n=3)	21.0	9.5	2.2	Comb-shaped, EC absent	3, 6
*G. tamakosi* (n=4)	18.6	9.3	2.0	Comb-shaped, EC absent	1
*Rishetia capillacea* (n=2)	10.5	3.5	3.0	Tubular sac, EC ?	1
*R. garoense* (n=2)	27.0	5.3	5.2	Tubular sac, EC ?	2
*R. hastula* (n=10)	11.5	3.8	3.0	Tubular sac, EC present	1
*R. kathmandica* (n=22)	33.3	8.6	3.9	Tubular sac, EC present	1
*R. longispira* (n=4)	39.7	9.3	4.3	Tubular sac, EC present	2
R. cf. mastersi (n=13)	14.7	5.5	2.7	Tubular sac, EC present	1
*R. nagarjunensis* (n=4)	36.5	11.1	3.3	Simple knob, EC present	1
*R. rishikeshi* (n=7)	13.2	5.0	2.7	Tubular sac, EC present	1
*R. subulata* (n=4)	11.9	3.8	3.1	Tubular sac, EC present	1
*R. tribhuvana* (n=7)	9.6	2.9	3.3	Tubular sac, EC present	1

References: 1 = present study, 2 = Godwin-Austen (1920), 3 = Godwin-Austen (1918), 4 = Fernando (1950), 5 = Semper (1877), 6 = Schileyko (1999), 7 = Pilsbry (1908–1909).

Finally, Schileyko (1999) recognised the Glessulidae Godwin-Austen, 1920 as a distinct family and introduced the Rishetiinae (with *Rishetia* as type genus) as a new subfamily within the Subulinidae. He also included the genera *Eutomopeas* Pilsbry, 1946, *Tortaxis* Pilsbry, 1906 and *Bacillum* Theobald, 1870 within Rishetiinae based on the presence of some form of truncation of the columella. It remains to be decided whether this classification (and separation) of *Glessula* and *Rishetia* will stand a phylogenetic analysis. This study illustrated the spermatophore of *R. hastula*, the first observation of a spermatophore in Glessulinae. This observation adds to the accumulating evidence that in contrast to Tompa’s (1984) suggestion that Achatinidae and their relatives do not produce spermatophores, these structures may be not uncommon in the Achatinoid clade of the Stylommatophora. Indeed, spermatophores have been reported in Achatinidae (Plummer 1975), Subulinidae (Baker 1945, Marcus 1968, Naggs 1994, Medeiros et al. 2013) and Streptaxidae (de Winter et al. 1999, Gerlach and van Bruggen 1999, de Winter and Vastenhout 2013, Rowson and Tattersfield 2013).

## Supplementary Material

XML Treatment for Glessula

XML Treatment for Glessula orobia

XML Treatment for Glessula cf. hebetata

XML Treatment for Glessula tamakoshi

XML Treatment for Rishetia

XML Treatment for Rishetia hastula

XML Treatment for Rishetia kathmandica

XML Treatment for Rishetia cf. mastersi

XML Treatment for Rishetia nagarjunensis

XML Treatment for Rishetia rishikeshi

XML Treatment for Rishetia subulata

XML Treatment for Rishetia tribhuvana
